# Influencing factors of short-form video addiction among Chinese university students: a systematic review

**DOI:** 10.3389/fpsyg.2025.1663670

**Published:** 2025-09-22

**Authors:** Xiaoling Zhan, Weijia Zhu

**Affiliations:** ^1^School of the Arts, Wuhan Business University, Wuhan, Hubei, China; ^2^School of Art, Wuhan University, Wuhan, Hubei, China

**Keywords:** short-form video addiction, Chinese university students, influencing factors, systematic review, social media addiction

## Abstract

**Introduction:**

This systematic review explores the influencing factors of short-form video addiction (SVA) among Chinese university students over the past 5 years. It offers theoretical and practical implications for understanding and preventing SVA among university students and suggests future research directions.

**Methods:**

This review identified 28 eligible peer-reviewed articles from seven English and Chinese databases. The protocol was pre-registered in PROSPERO (CRD420251030636).

**Results:**

The findings indicate that SVA among university students is prevalent and multifactorial. Influencing factors include eight domains: demographic, psychological, personality traits, behavioral, social, family, motivation for media use, and platform-induced factors.

**Discussion:**

Guided by the I-PACE model, the review organized these domains into the person–affect–cognition–execution framework. Personal factors include demographic and personality traits; social and family factors represent persons' external environmental influences; affective and cognitive components comprise psychological factors and media use motivations; and execution factors include behavioral and platform-induced influences. These components interact with each other and collectively predict SVA throughout university students' development. Psychological and personality trait factors often serve as mediating or chained mediating variables for other influencing factors. Conflicting findings regarding demographic, personality, and psychological influences may result from sample homogeneity and research design limitations. Research gaps remain in policy factors and longitudinal or intervention-based studies.

**Systematic review registration:**

https://www.crd.york.ac.uk/prospero/display_record.php?ID=CRD420251030636, PROSPERO: CRD420251030636.

## 1 Introduction

With the development of the internet, short-form video addiction (SVA) has attracted more attention from mass media and researchers (Ye et al., 2022). Short-form videos refer to videos displayed on online platforms for people to watch and share at any time, and the length of time is usually measured in seconds, ranging from a few seconds to 6 min (Wu et al., 2021). It combines music, images, and text (Wu et al., 2021). Short-form video, one of the most popular and widely distributed video products on the internet, has accelerated the speed of information dissemination (Huangfu et al., 2022). It caters to people's fast-paced lifestyles and presents exciting content to viewers in a short time (Chen Y. et al., 2023). However, as a new and popular social media with distinctive features, short-form videos may be more likely to lead to addiction and bring negative impacts to users. The problem of SVA in China has increased since the COVID-19 pandemic, and the average daily use of short-form video apps in China has grown to 600 million hours (Dai et al., 2021). In 2020, there were nearly 850 million online video users in China, among whom 773 million were short-form video users, with a user utilization rate of 85.6% (Chung, 2022). SVA threatens users' physical and mental health (Zhang et al., 2019). Therefore, SVA has become a critical topic of great interest and has attracted many studies from multiple academic subjects (Ye et al., 2022).

SVA refers to the excessive and irrational watching of short-form videos and the negative impact on daily life (Ye et al., 2022). Short-form video duration, rich content, personalized recommendations, immersive experience, and fragmented pattern may be more likely to lead to user addiction (Meng and Leung, 2021). Addiction is a state that fundamentally maintains specific behaviors. It is a subconscious phenomenon, a behavioral factor that excludes compulsion (Khantzian, 2003). Short-form video applications can send customized content based on user preference analysis; users can also choose to watch their favorite specific creators and content. The fragmentation pattern effectively increases the speed of information dissemination and enables users to browse content of interest quickly. However, the fragmentation pattern can strongly stimulate the brain's pleasure center in a short time (Tian et al., 2023) and lead to a significant release of dopamine (Wise and Robble, 2020). Continued exposure to intense pleasure and large amounts of dopamine leads to more profound addiction and more significant cravings in the user. It reduces dopaminergic enzyme activity and the availability of dopamine transporter proteins, a classic addiction symptom (Akgun and Mirzajee, 2024).

Among the vast number of Chinese short-form video users, 89.7% are highly educated Chinese university students between 20 and 29 (Yang et al., 2021). Therefore, university students face a high risk of addiction as the primary users of short-form video applications (Liu et al., 2021). They prefer straightforward video content of shorter duration (Violot et al., 2024) and are more likely to spend most of their time on short clips. It would result in lower grades (Ye et al., 2022), distraction, and poor time management (Chen Y. et al., 2023). Excessive use of digital social media may affect the normal development of the adolescent brain and even impair attention, memory, and learning abilities (Farchakh et al., 2020). At the same time, SVA brings many psychological problems to university students (Ye et al., 2022). Consequently, exploring SVA among Chinese university students has significant theoretical and practical implications.

However, fewer research findings exist on SVA among Chinese university students. It is even more of a research gap for a comprehensive and systematic review to identify future research directions. In order to fill this gap, this study investigated the factors that influence SVA among Chinese university students. It analyses research trends over the past 5 years. Furthermore, this review identifies gaps in current relevant research and provides suggestions for future research.

This systematic review examined the factors influencing SVA among Chinese university students over the last 5 years (2020–2025). The included articles were from 7 databases, both in Chinese and English, totaling 28 articles. Guided by the PICOS framework, we extracted information on population, intervention or exposure, outcomes, and study design. Most studies lacked a formal control group, so we did not include a comparison component. To synthesize the findings, we adopted the person-affect-cognition-execution (I-PACE) interaction model (Brand et al., 2019) to discuss the factors influencing SVA among Chinese university students. This model suggests that addictive behaviors arise from the interaction between a person's core characteristics (the “person”), their emotional and cognitive responses (the “affect” and “cognition”), and behavior (the “execution”). This model provides a clear structure for organizing the multifaceted influences on SVA and has been widely applied to research on problematic internet use (Schimmenti, 2023).

## 2 Materials and methods

### 2.1 Protocols and registrations

This review used the Preferred Reporting Items for Systematic Reviews and Meta-Analyses (PRISMA) guidelines. The protocol was pre-registered in PROSPERO (CRD420251030636).

### 2.2 Search strategy and procedure

**Figure 1:**
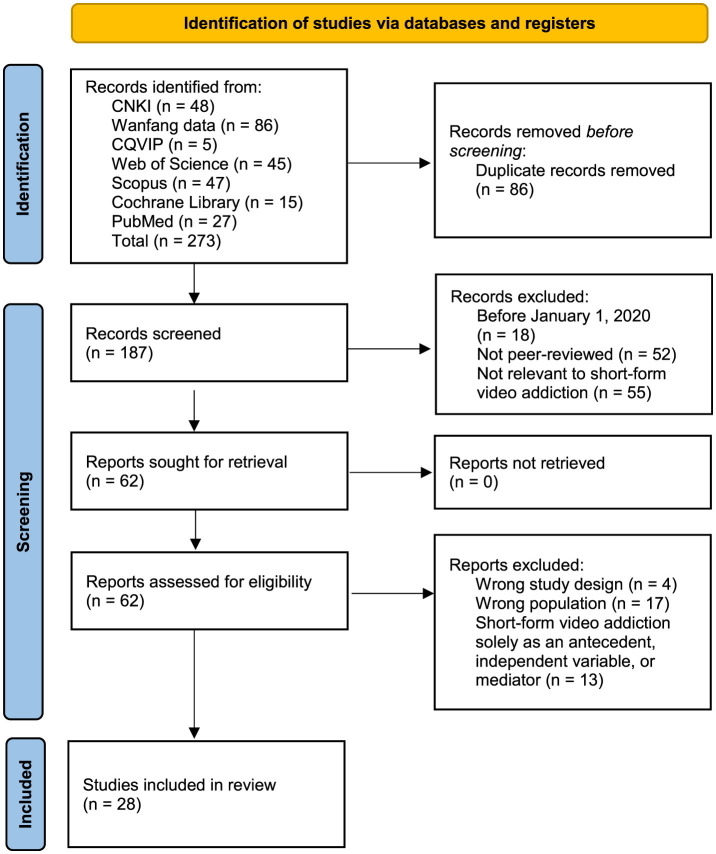
PRISMA flow diagram.

This article uses a search algorithm to systematically search seven electronic databases, including China National Knowledge Infrastructure (CNKI), Wanfang Data, CQVIP, Web of Science (WoS), Scopus, the Cochrane Library, and PubMed. The search algorithm included the following relevant keywords: “short-form video” or “short video” or “Douyin” or “Kuaishou” or “short video application,” and “addiction” or “problematic use” or “dependence” or “overuse” or “indulgence,” and “Chinese college student” or “Chinese university student” or “Chinese undergraduate” or “college student” or “university student” or “undergraduate.” The support material presents the specific search strategies for each database. The flowchart in Figure 1 illustrates the procedure for selecting studies for the analysis.

### 2.3 Eligibility criteria

Inclusion criteria: (1) Research design: cross-sectional, intervention or experimental, and longitudinal study were included to capture both correlational and causal evidence; (2) Study population: Chinese university students, as this demographic shows the highest SVA prevalence; (3) Study focus: studies that investigate the influencing factors of SVA, with SVA conceptualized as the outcome or dependent variable. Studies must report at least one correlation between SVA and other variables or influencing factors; (4) Country/Region: mainland China; (5) Article type: peer-reviewed article; (6) Time frame: published between 1 January 2020 and 6 July 2025; (7) Language: English and Chinese.

Exclusion criteria: (1) Article type only reviews, press releases, commentaries, thesis, and conference announcements; (2) Full text unavailable; (3) Insufficient methodological or statistical detail preventing data extraction; (4) Published before 1 January 2020; (5) Studies that conceptualize SVA solely as an antecedent, independent variable, or mediator.

### 2.4 Research quality assessment

This review used the Mixed Methods Appraisal Tool (MMAT) and its user guide to assess research quality comprehensively (Hong et al., 2018). The MMAT evaluates qualitative, quantitative, and mixed-method studies, making it suitable for systematic reviews with diverse methodologies. It begins with two screening questions: (1) Are the research questions clear? (2) Do the collected data allow the research questions to be addressed? All studies in this review met both criteria. We then applied the MMAT to assess the methodological quality of each study design. For each criterion, we recorded responses as “yes,” “no,” or “unable to say,” without calculating a total score, as scoring was not our primary aim. Table 1 presents the quality assessment for each criterion and study. Two researchers independently conducted the assessments, and a third researcher resolved disagreements.

**Table 1:** Quality evaluation of the included studies.

**No**.	**Study**	**Screening S1**	**Screening S2**	**1.1**	**1.2**	**1.3**	**1.4**	**1.5**	**4.1**	**4.2**	**4.3**	**4.4**	**4.5**	**5.1**	**5.2**	**5.3**	**5.4**	**5.5**
1	Ding et al. (2024)	Y	Y						Y	Y	Y	Y	Y					
2	Fu et al. (2024)	Y	Y						Y	Y	Y	Y	Y					
3	Liu et al. (2025)	Y	Y						Y	Y	Y	Y	Y					
4	Liu et al. (2021)	Y	Y						Y	Y	Y	Y	Y					
5	Pan et al. (2024)	Y	Y						Y	Y	Y	Y	Y					
6	Wang and Zhang (2024)	Y	Y						Y	Y	Y	Y	Y					
7	Wu et al. (2024)	Y	Y						Y	Y	Y	Y	Y					
8	Xu et al. (2025)	Y	Y						Y	Y	Y	Y	Y					
9	Xue et al. (2025)	Y	Y						Y	Y	Y	Y	Y					
10	Xue et al. (2024)	Y	Y						Y	Y	Y	Y	Y					
11	Yang et al. (2025)	Y	Y						Y	Y	Y	Y	Y					
12	Yao et al. (2025)	Y	Y						Y	Y	Y	Y	Y					
13	Yue et al. (2024)	Y	Y						Y	Y	Y	Y	Y					
14	Zhang L. et al. (2024)	Y	Y						Y	Y	Y	Y	Y					
15	Zhang D. et al. (2023)	Y	Y						Y	Y	Y	Y	Y					
16	Zhang Y. et al. (2024)	Y	Y						Y	Y	Y	Y	Y					
17	Zhang et al. (2025)	Y	Y						Y	Y	Y	Y	Y					
18	Zhao and Kou (2024)	Y	Y						Y	Y	Y	Y	Y					
19	Hang (2020)	Y	Y						Y	Y	Y	Y	Y					
20	Yawen et al. (2020)	Y	Y						Y	Y	Y	Y	Y					
21	Qingqing and Liqin (2021)	Y	Y						Y	Y	Y	Y	Y					
22	Quanxi et al. (2024)	Y	Y						Y	Y	Y	Y	Y					
23	Ling et al. (2021)	Y	Y						Y	Y	Y	Y	Y					
24	Yue et al. (2022)	Y	Y						Y	Y	Y	Y	Y					
25	Jianya et al. (2020)	Y	Y						Y	Y	Y	Y	Y					
26	Pei (2022)	Y	Y	Y	Y	Y	Y	Y										
27	Li et al. (2022)	Y	Y						Y	Y	Y	Y	Y					
28	Chen X. et al. (2023)	Y	Y											Y	Y	Y	Y	Y

S1: Are the research questions clear? S2: Do the collected data allow the research questions to be addressed?

1. Qualitative 1.1. Is the qualitative approach appropriate to answer the research question? 1.2. Are the qualitative data collection methods adequate to address the research question? 1.3. Are the findings adequately derived from the data? 1.4. Is the interpretation of results sufficiently substantiated by data? 1.5. Is there coherence between qualitative data sources, collection, analysis, and interpretation?

4. Quantitative descriptive 4.1. Is the sampling strategy relevant to address the research question? 4.2. Is the sample representative of the target population? 4.3. Are the measurements appropriate? 4.4. Is the risk of non-response bias low? 4.5. Is the statistical analysis appropriate to answer the research question?

5. Mixed methods 5.1. Is there an adequate rationale for using a mixed methods design to address the research question? 5.2. Are the different components of the study effectively integrated to answer the research question? 5.3. Are the outputs of the integration of qualitative and quantitative components adequately interpreted? 5.4. Are divergences and inconsistencies between quantitative and qualitative results adequately addressed? 5.5. Do the different components of the study adhere to the quality criteria of each tradition of the methods involved?

N, no; Y, yes.

All included studies scored seven on the quality assessment (see Table 1), indicating sound design and execution, with outcome measures and statistical analyses showing high validity and reliability. Although we did not perform formal GRADE evidence grading due to heterogeneity in study designs, we qualitatively evaluated the methodological quality, findings' consistency, and evidence's directness. We did not conduct a formal risk of bias analysis because most studies were cross-sectional, but we identified potential sources of bias. We could not conduct a meta-analysis due to variations in study designs, measures, and reported outcomes. Instead, we performed a narrative synthesis, grouping the findings into eight factor categories.

### 2.5 Data extraction

We produced a data extraction form to record relevant data from the included studies. Based on the PICOS framework, our data extraction included population: sample size, research area, and gender (male/female). Intervention (exposure): influencing factor categories and the short-form video application. Comparator: differing levels or categories of exposures, but no formal control group in most included studies. Therefore, this review did not extract a control group. Outcome: main findings. Study design: SVA measurement, research design, and research methodology. This structure enhances transparency and facilitates replication of the review process.

## 3 Results

### 3.1 Descriptive analysis

This systematic review finally included 28 articles for analysis, including English (*n* = 19) and Chinese (*n* = 9). The results of the extracted data are illustrated in Table 2.

**Table 2:** Data extraction.

**Study**	**Sample size**	**Male/ female**	**Research area**	**Research design**	**Research methodology**	**SVA measurement**	**Short-form video application**	**Influencing factor categories**	**Main findings**
Ding et al. (2024)	694	311/383	Mainland China	Cross-sectional	Quantitative	SVA scale for college students	N	Personality trait, psychological, social	Low subjective wellbeing (*M* = −0.004), extraverted personality (*M* = −0.013), core self-evaluation (*M* = −0.007), and social support (*M* = −0.011) are more likely to engage in high-risk SVA behaviors to balance their cognition
Fu et al. (2024)	1,187	429/758	Mainland China	Cross-sectional	Quantitative	SVA scale for college students	N	Family, psychological	Reciprocal filial piety significantly and negatively predicted SVA (B = – 0.204, *P* < 0.001). Authoritarian filial piety significantly and positively predicted SVA (*B* = 0.214, *P* < 0.001). Interaction of intolerance of uncertainty and self-control significantly predicted SVA (*B* = – 0.075, *t* = – 3.781, *P* < 0.001)
Liu et al. (2025)	2,239	948/1,291	Guangxi province, Yunnan province, Guangzhou city, and Wuhan city, China	Cross-sectional	Quantitative	SVA scale	N	Psychological, demographic, behavioral	Students with excessive past and present time focus were more likely to fall into the high-risk SVA category (*r* = 0.13, *P* < 0.01; *r* = 0.11, *P* < 0.01). Inhibitory control is significantly positively correlated with SVA (*r* = 0.25, *P* < 0.01), whereas initiation control is negatively correlated with SVA (*r* = −0.64, *P* < 0.01). Female students were more likely than male students to fall into the low (male: OR = −1.19, *P* < 0.001; CI = 0.23–0.41), moderate (OR = −0.66, *P* < 0.01; CI = 0.39~0.69), or high addiction (OR = −1.79, *P* < 0.01; CI = 0.11–0.25) categories. Only children were more likely to belong to the moderate (OR = 0.43, *P* < 0.05; CI = 0.46–0.93) or high-risk addiction categories (OR = 0.62, *P* < 0.05; CI = 0.45–0.93) than non-only children
Liu et al. (2021)	990	474/516	Shandong province, China	Cross-sectional	Quantitative	6-items SVA questionnaire	N	Motivation for media use, personality trait, psychological	Perceived stress was positively associated with SVA (total effect = 0.422, *P* < 0.001, 95% CI = 0.347–0.473), and self-compensation motivation partially mediated this association (Indirect effect = 0.045, 95% CI = 0.027–0.063). Shyness moderates the direct effect of perceived stress (β = 0.076, *t* = 2.554, *P* < 0.01, CI = 0.018–0.134) and the latter part of the mediating effect of self-compensation motivation (β = 0.117, *t* = 3.952, *P* < 0.001, CI = 0.059–0.176)
Pan et al. (2024)	1,386	417/969	Anhui province, China	Cross-sectional	Quantitative	SVA scale	N	Demographic, motivation for media use	Gender (male: β = −0.89, OR = 0.41), major type (art and human sciences: β = 0.28, OR = 1.33; natural science: β = 0.06, OR = 1.06), and family location (urban: β = −0.08, OR = 0.92; rural: β = −0.68, OR = 0.51) were associated with among Chinese university students' SVA (all *P* < 0.05)
Wang and Zhang (2024)	664	195/469	Shaanxi province, China	Cross-sectional	Quantitative	4-items SVA questionnaire	TikTok (78.16%), Beili and Xiaohongshu (34.49%), Weibo (21.23%), Kuaishou (18.37%)	Psychological, motivation for media use, platform-induced	Influencing factors for SVA are an individual's poor self-control, content's satisfying self-needs, and the platform's self-preference
Wu et al. (2024)	560	225/335	Jiangsu province, Zhejiang province, Yunnan province, Shanghai city, and Chongqing city, China	Cross-sectional	Quantitative	SVA scale	N	Psychological, personality trait	Proactive personality negatively predicts SVA (*r* = −0.408, *P* < 0.01). Moreover, both resilience (β = −0.063, *P* < 0.01) and self-control (β = −0.217, *P* < 0.001) acted as independent mediators, as well as forming a significant serial mediation pathway (β = −0.040, *P* < 0.001) in the relationship between proactive personality and SVA
Xu et al. (2025)	563	283/280	Hangzhou Normal University, China	Cross-sectional	Quantitative	8-items problematic short-video use questionnaire	N	Psychological, personality trait	Positive association between adult attention deficit hyperactivity disorder (ADHD) symptoms (β = 0.34, *P* < 0.001) and problematic short-video use (PSVU). A serial mediating effect of cognitive reappraisal, emotional distress, and boredom proneness for the association between ADHD and PSVU (Estimate = 0.005, 95% CI = 0.001–0.014). Both specifically inattention (Estimate = 0.055, 95% CI = 0.021–0.099) and hyperactivity-impulsivity (Estimate = 0.088, 95% CI = 0.043–0.150) symptoms influence PSVU with increased emotional distress and boredom proneness
Xue et al. (2025)	11,425	5,973/5,452	Wuhan city, China	Cross-sectional	Quantitative	20-items SVA questionnaire	TikTok (72.19%); Tencent Weishi; Kuaishou; Huoshan Video; Haokan Video; Pear Video; Baidu	Family, psychological, demographic, social	A significant increase in the likelihood of SVA as the number of adverse childhood experiences (ACEs) increased (OR: 2.40, 95% CI = 2.001–2.888; OR: 4.68, 95% CI = 3.467–6.325). A strong linear association was observed between child abuse/neglect (OR: 1.49, 95% CI = 1.388–1.604), violence outside the family (OR: 1.30, 95% CI = 1.170–1.449), and SVA. Furthermore, significant differences were found based on sex (OR: 1.46, 95% CI = 1.345–1.595), grade (OR: 1.89, 95% CI = 1.509–2.367), and major (OR: 1.47, 95% CI = 1.266–1.699). Resilience and life satisfaction may serially mediate the relationship between ACEs and SVA (β = 0.009, SE = 0.001, 95% CI = 0.006–0.011)
Xue et al. (2024)	13,307	7,129/6,178	North, South, West, and Central China	Cross-sectional	Quantitative	20-items SVA questionnaire	N	Family, psychological	Compared to participants solely separated from parents (*M* = 32.32, SE = 0.54, *P* < 0.001), those exposed to abuse and neglect (*M* = 38.74, SE = 0.93, *P* < 0.001) in the “high violence and left behind” category demonstrate more pronounced addiction susceptibilities
Yang et al. (2025)	85	N	Mainland China	Cross-sectional	Quantitative	Short-form video app addiction scale	N	Behavioral	Physical activity was directly associated only with reduced nighttime short video usage (β = −0.12; *P* < 0.05). Additionally, physical activity was indirectly associated with reduced short video usage during nighttime (β = −0.03; *P* < 0.05), study time (β = −0.03; *P* < 0.05), and leisure time (β = −0.04; *P* < 0.05), as well as lower levels of SVA (β = −0.06; *P* < 0.05), mediated by depression
Yao et al. (2025)	Study 1: 1,615 (total, *n* = 2,884) Study 2: 112	558/1,057 68/44	Mainland China	Cross-sectional	Quantitative	Smartphone addiction scale short revision (SAS-SV)	N	Social, platform-induced, psychological	Study 1: Significant correlations between SVA and bullying victimization (*r* = 0.363, *P* < 0.001), including subdimensions such as verbal (*r* = 0.338, *P* < 0.001), physical (*r* = 0.288, *P* < 0.001), and relational bullying (*r* = 0.356, *P* < 0.001), as well as negative affect (*r* = 0.291, *P* < 0.001). Negative affect (indirect effects = 0.057, 95% CI = 0.038–0.081, mediation effect accounted for 18.15%) partially mediated the relationship between bullying victimization and SVA. Study 2: SVA was positively correlated with amplitude of low-frequency fluctuations in the inferior temporal gyrus and parahippocampus. These neural circuits related to reward processing, attention grabbing, and emotion regulation on short video platforms.
Yue et al. (2024)	336	139/197	Mainland China	Cross-sectional	Quantitative	SVA Scale	N	Psychological, social	Negative cognitive bias (*r* = 256, *P* < 0.01) was positively correlated with SVA. Social support (*B* = 0.054, 95% CI = 0.021–0.095) and loneliness (*B* = 0.107, 95% CI = 0.053–0.173) separately mediated the relationship between negative cognitive bias and SVA. Social support (*B* = 0.043, 95% CI = 0.129–0.081) and loneliness (*B* = 0.086, 95% CI = 0.040–0.142) sequentially mediated the relationship between negative cognitive bias and SVA
Zhang L. et al. (2024)	804	287/517	Mainland China	Cross-sectional	Quantitative	14-items college students' SVA questionnaire	N	Personality trait, psychological	Neuroticism (β = 0.569, *P* < 0.001), agreeableness (β = 0.141, *P* < 0.05), and extraversion (β = 0.238, *P* < 0.001) can predict SVA. Both neuroticism (indirect effect = 0.068, 95% CI = 0.012–0.120) and agreeableness (indirect effect = 0.227, 95% CI = 0.101–0.354) can first induce depression and then lead to anxiety and SVA
Zhang D. et al. (2023)	331	152/179	Xi'an city, China	Longitudinal	Quantitative	Short video usage behavior scale	N	Personality trait, psychological	A significant positive correlation between short video overuse behavior and various dimensions of depression in both tests. The correlation coefficient between the two short video overuse behaviors is 0.64. Short video overuse behavior significantly and positively predicted depression (β = 0.182, SE = 0.047, *P* < 0.001); however, depression did not significantly predict short video overuse behavior (β = 0.031, SE = 0.049, *P* > 0.05).
Zhang Y. et al. (2024)	523	206/317	Shandong province, China	Cross-sectional	Quantitative	Social media addiction scale	N	Social, psychological, personality trait	Social exclusion (*M* = 2.32, SD = 0.81) was found to positively predict SVA. The relationship was significantly mediated by boredom (Indirect effect = 0.04, 95% CI = 0.02–0.07) and self-control (Indirect effect = 0.04, 95% CI = 0.01–0.07). A significant chained mediation through boredom and self-control was also identified
Zhang et al. (2025)	104	21/83	Mainland China	Experimental intervention	Quantitative	Smartphone addiction scale-short version, mobile phone type addiction scale	N	Behavioral	The experimental group receiving brief online mindfulness-based intervention showed a significant decrease in SVA, *f*_(1, 99)_ = 114.64, *P* < 0.001, η^2^/*p* = 0.537 at the post-test level compared to the pre-test. Trait mindfulness (β = −0.149, *P* < 0.01 = 0.007) negatively predicted SVA.
Zhao and Kou (2024)	388	121/267	Central China	Cross-sectional	Quantitative	College student SVA scale	N	Psychological, behavioral, social	Loneliness (*r* = 0.507, *P* < 0.01) significantly positively affected SVA. The association between loneliness and short video addiction was independently mediated by social support (11.86%) or physical activity (5.52%). Social support and physical activity play a chain mediating role (3.27%) in the association between loneliness and short video addiction
Hang (2020)	282	126/156	Shaanxi Normal University, Xi'an University of Posts and Telecommunications, and Northwest University of Politics and Law, China	Cross-sectional	Quantitative	3-items mobile phone entertainment addiction tendency questionnaire	TikTok	Psychological, behavioral, social	University students' tendency to indulge in mobile entertainment is closely related to their anxiety, depression, and interpersonal sensitivity factors. There was a significant correlation between university students' fondness for handheld games and their depression factor (*P* < 0.05), the length of time spent watching TikTok and its anxiety factor was extremely significant (*P* < 0.001), and fondness for TikTok was significantly correlated with its interpersonal relationship sensitivity factor (*P* = 0.012 < 0.05)
Yawen et al. (2020)	559	173/386	Mainland China	Cross-sectional	Quantitative	Revised Chen Internet Addiction Scale (CIAS-R)	N	Behavioral, demographic, personality trait	The female college students using short videos were significantly more than the male college students (χ^2^ = 4.389, *P* < 0.05). The total score of internet addiction (*t* = −2.761, *P* < 0.01), compulsive internet access (*t* =(*t* = −2.434, *P* < 0.05), with drawal response (*t* = −3.667, *P* < 0.001), tolerance (*t* = −3.716, *P* < 0.001) of college students with high short video frequency were significantly higher than those with low short video frequency, and the score of essence dimension was significantly lower than that of college students with low frequency (*t* = 2.081, *P* < 0.05). The scores of time management (*t* = −2.026, *P* < 0.05) and mental quality (*t* = −3.626, *P* < 0.001) of the students who used the short video for a long time were significantly higher than those of the students who used the short video for a short time, and the scores of cover up dimension were significantly lower than those of the students who used the short video for a long time (*t* = 1.975, *P* < 0.05). The frequency of introversion and extraversion of short video and neuroticism had a good predictive effect on inter- net addiction at the significant level of *P* = 0.01
Qingqing and Liqin (2021)	224	53/171	Mainland China	Cross-sectional	Quantitative	5-items Audience Dependency Questionnaire for Weibo, WeChat, and TikTok APPs	TikTok	Behavioral, demographic, motivation for media use, psychological	TikTok usage time (β = 0.394, *P* < 0.05), motivation (β = 0.196, *P* < 0.05), and psychology (β = 0.245, *P* < 0.05) were positively correlated with TikTok dependence, while frequency (β = 0.129, *P* > 0.05) and functional factors (β = 0.001, *P* > 0.05) were not strongly correlated with dependence.
Quanxi et al. (2024)	288	125/163	Wuhan city, China	Cross-sectional	Quantitative	3-items Mobile video addiction tendency questionnaire	N	Psychological, demographic, behavioral, social	Depression (β = 0.400, *P* < 0.01) and anxiety (*r* = 0.472, *P* < 0.01) were positively correlated with SVA. Learning engagement (*r* = −0.224, *P* < 0.01), interpersonal satisfaction (*r* = −0.252, *P* < 0.01), meaning in life (*r* = −0.105, *P* < 0.01), and academic achievement (*r* = −0.283, *P* < 0.05) were negatively correlated with mobile short video addiction. Meaning in life (β = 1.20, *P* < 0.01; β = −0.64, *P* < 0.01) partially mediated the relationship between learning engagement and SVA, with between academic achievement and SVA, respectively. Anxiety (β = −0.27, *P* < 0.01) and depression (β = 0.70, *P* < 0.01) plays a chain mediating role between interpersonal relationship satisfaction and short video addiction
Ling et al. (2021)	394	77/317	Shandong province, China	Cross-sectional	Quantitative	TikTok addiction scale for university students	TikTok	Personality trait, behavioral, psychological, motivation for media use, family, social	Second-year students (*M* = 98.27, *P* < 0.001) had higher SVA scores than first-year students. Those who used TikTok for 2–5 h per day (*M* = 220.90, *P* < 0.001) vs. more than 5–8 hours per day (*M* = 268.50, *P* < 0.001) had significantly higher scores than those who used TikTok for less than 2 h. High life stress (*M* = 206.55, *P* < 0.001), gaining more affirmation and satisfaction (*M* = 308.71, *P* < 0.001), soothing negative emotions (*M* = 212.60, *P* < 0.001), surrounding people's use (*r* = 195.22, *P* < 0.001); personality that was active (*r* = 0.111, *P* < 0.05), straightforward (*r* = 0.148, *P* < 0.05), altruistic (*r* = 0.102, *P* < 0.05), and authoritarian parenting were positively associated with SVA. Emotion-oriented personality (*r* = 0.135; *r* = 0.234, *P* < 0.05), and permissive parenting were associated with low and healthy SVA. The conscientious (*r* = −0.209), and agreeableness (*r* = −0.160) personality was negatively associated with SVA inefficacy (*P* < 0.05)
Yue et al. (2022)	351	105/246	Guangzhou city, Kunming city, China	Cross-sectional	Quantitative	9-items TikTok addiction questionnaire	TikTok	Psychological, social, family	The report rate of university students' TikTok dependency was 20.51%. University students who had a positive cognitive of TikTok were positively correlated with TikTok dependency (OR = 1.1, 95% CI = 1.02–1.22, *P* < 0.05); behavior pressure of friends (OR = 3.27, 95% CI = 1.33–8.03, *P* < 0.01) and family (OR = 2.21, 95% CI = 1.05–4.67, *P* < 0.05; OR = 3.41, 95% CI = 1.50–7.57, *P* < 0.01) was positively correlated with TikTok dependency
Jianya et al. (2020)	479	228/251	Mainland China	Cross-sectional	Quantitative	Short video overuse scale	N	Social, psychological, motivation for media use	Peer influence (β = 0.187, *P* < 0.001), pressure relief (β = 0.303, *P* < 0.001), amusement (β = 0.130, *P* < 0.001), escapism (β = 0.214, *P* < 0.001), and information acquisition (β = 0.117, *P* = 0.021) have significant positive effect on mobile video overuse behavior of college students, while self-control (β = −0.184, *P* < 0.001) and media literacy (β = −0.121, *P* < 0.001) have significant negative effect on it
Pei (2022)	10	4/6	Mainland China	Cross-sectional	Qualitative	Interview	N	Psychological, motivation for media use	SVA is closely related to emotional expression in real life, identity in the virtual world, and unrealistic fantasies of Internet celebrities.
Li et al. (2022)	1,035	358/677	North, South, West, and Central China	Cross-sectional	Quantitative	10-items Smartphone addiction questionnaire,	N	Behavioral	Short videos (48.26%) are the second most common purpose for using a smartphone before bed. Watching short videos (>60%) and playing games are more likely to lead to mobile phone addiction behavior before bedtime
Chen X. et al. (2023)	Study 1: 183 Study 2: 10	Study 1: 90/93 Study 2: 5/5	Beijing Institute of Graphic Communication, Zhejiang University, China	Cross-sectional	Mixed	6-items Smartphone usage questionnaire, and interview	WeChat video, TikTok, Kuaishou, iQIYI, Youku, Bilibili, YouTube	Platform-induced	Social network and short video apps were the most mentioned apps in the questionnaire, occupying much of the interviewees' screen time. Persuasive short-video applications include TikTok and Kuaishou. Negative sentiment is associated with TikTok (spending too much time on TikTok, “cannot stop”). Short videos, user-generated content, and streaming platforms recommend new videos/playlists based on users' viewing history. TikTok and Kuaishou integrate buying links with video content, encouraging users to place orders with only one click. Persuasive designs are making smartphones more addictive

N/A, no answer; P, P value; B, unstandardized regression coefficient; OR, odds ratio; CI, confidence interval; β, beta coefficient; M, mean; SD, standard deviation; SE, standard error; t, t-test value; f, analysis of variance value; χ^2^, chi-square test value; η^2^/*p*, Partial Eta Squared.

The research areas included the following regions: Guangxi Province (*n* = 1), Yunnan Province (*n* = 2), Shandong province (*n* = 3), Anhui province (*n* = 1), Guangzhou city (*n* = 2), Wuhan City (*n* = 3), Shaanxi Province (*n* = 1), Shaanxi Normal University (*n* = 1), Jiangsu province (*n* = 1), Zhejiang Province (*n* = 1), Zhejiang University (*n* = 1), Hangzhou Normal University (*n* = 1), Kunming city (*n* = 1); Shanghai city (*n* = 1), Chongqing city (*n* = 1), North (*n* = 2), South (*n* = 2), Central (*n* = 3), and West, China (*n* = 2), Xi'an city (*n* = 1), Xi'an University of Posts and Telecommunications (*n* = 1), Northwest University of Politics and Law (*n* = 1), Beijing Institute of Graphic Communication (*n* = 1), Mainland China (*n* = 11).

Most articles used a cross-sectional research design (*n* = 26) and relied on subjective self-reporting. Only Zhang et al. (2025) used an experimental intervention, and Zhang D. et al. (2023) used a longitudinal study as the study design. The majority of articles adopted a quantitative research methodology (*n* = 26), with only Pei (2022) employing qualitative and Chen X. et al. (2023) using mixed research methodology. The literature that reported sample sizes (*n* = 27) usually had more female students than male students (*n* = 24). SVA measurement includes scales (*n* = 15), questionnaires (*n* = 12), and interviews (*n* = 2). The scales focused on the SVA scale (*n* = 12), but also included the Smartphone Addiction Scale (*n* = 2), Social Media Addiction Scale (*n* = 1), and the Internet Addiction Scale (*n* = 1). Among the articles reporting on short-form video applications (*n* = 7), TikTok was the most widely used platform by Chinese university students.

Influencing factors of SVA among Chinese university students have centered on eight categories: demographic, psychological, personality trait, behavioral, social, family, motives for media use, and platform-induced factors.

### 3.2 Demographic factors

Demographic factors (*n* = 6) influencing SVA among Chinese university students, including gender (*n* = 5), only child (*n* = 1), grade (*n* = 3), major type (*n* = 3), and family location (*n* = 1). The findings indicated that the percentage of female university students with SVA is significantly higher than that of male university students (Liu et al., 2025; Pan et al., 2024; Xue et al., 2025; Yawen et al., 2020). However, two studies argue that males may be more engaged with short-form videos and more likely to have moderate to severe SVA (Xue et al., 2025; Yue et al., 2022). Students born in non-only-child families are more at risk of SVA than those born in only-child families (Liu et al., 2025). Students majoring in arts and humanities were 1.22–1.47 times more likely to have mild and moderately severe SVA than students majoring in science, technology, engineering, and mathematics (Xue et al., 2025). Medical students are more likely to become addicted (Yue et al., 2022). Compared to students in arts and sports, students in literature and history are less SVA (Pan et al., 2024). Students from rural areas are more likely to be at risk of high SVA than urban students (Pan et al., 2024). As the academic year increases, the likelihood of SVA decreases (Pan et al., 2024; Xue et al., 2025). However, one study concluded that third-year students are at a higher risk of SVA than first-year students, since they face a job hunt and need to communicate with companies (Quanxi et al., 2024).

### 3.3 Psychological factors

Psychological factors (*n* = 21) are an important factor influencing SVA among Chinese university students. The articles included in this review indicate that psychological factors include self-control (*n* = 6), subjective wellbeing (*n* = 2), stress (*n* = 2), emotional distress (*n* = 10), cognitive (*n* = 4), resilience (*n* = 1), and meaning in life (*n* = 1). Emotional distress refers to the negative effects of depression, anxiety, and loneliness that directly result in SVA (Hang, 2020; Quanxi et al., 2024; Wu et al., 2024; Xu et al., 2025; Xue et al., 2024; Yao et al., 2025; Yue et al., 2024; Zhang L. et al., 2024; Zhao and Kou, 2024). However, one study argued that depression does not significantly predict SVA (Zhang D. et al., 2023). University students with high life stress (Ling et al., 2021; Liu et al., 2021), high levels of meaning in life (Quanxi et al., 2024), low subjective wellbeing, including low life satisfaction (Ding et al., 2024; Xue et al., 2025), positively predict SVA. On the cognitive dimension, intolerance of uncertainty, negative cognitive biases, and reduced cognitive reappraisal due to adult ADHD can contribute to SVA (Fu et al., 2024; Xu et al., 2025; Yue et al., 2024). Instead, students with strong proactive self-control are less likely to be at high risk for SVA (Fu et al., 2024; Jianya et al., 2020; Liu et al., 2025; Wang and Zhang, 2024; Wu et al., 2024; Zhang Y. et al., 2024). Students with resilience and positive cognitive abilities are less prone to SVA (Wu et al., 2024; Yue et al., 2022).

### 3.4 Personality trait factors

Thus far, studies report conflicting results on the relationship between personality trait factors (*n* = 7) and the SVA. Most of the studies concluded that the personality traits of neuroticism (*n* = 2), shyness (*n* = 1), introversion (*n* = 1), boredom (*n* = 2), and low core self-evaluation (*n* = 1) positively predicted SVA. Chinese university students with higher levels of neuroticism, shyness, and introversion may be more at risk for interpersonal alienation, depression, and anxiety (Liu et al., 2021; Yawen et al., 2020; Zhang L. et al., 2024). Individuals with low core self-evaluation typically have a negative self-concept (Ding et al., 2024). In order to escape face-to-face communication, real-world discomfort, and the pursuit of defensive goals, students with the above personality trait tend to integrate into the online environment, resulting in SVA (Liu et al., 2021; Yawen et al., 2020; Zhang L. et al., 2024). Although boredom can directly lead to SVA, the included articles usually discuss it as a moderating or chain-moderating effect. For example, boredom mediated the effects of social exclusion and ADHD on SVA (Zhang Y. et al., 2024).

In contrast, the personality traits of agreeableness (*n* = 1), proactive (*n* = 1), extraverted (*n* = 3), conscientious (*n* = 1), emotion-oriented (*n* = 1) are less prone to SVA. Higher agreeableness may reduce the impact of negative emotions and thus reduce the risk of SVA (Zhang L. et al., 2024). Individuals with proactive personalities may be able to balance work and life more effectively to avoid overwork, thus reducing the risk of addiction (Wu et al., 2024). The extraverted, conscientious, and emotion-oriented personality is less likely to lead to SVA or to influence lower levels of SVA (Ding et al., 2024; Yawen et al., 2020; Zhang L. et al., 2024). However, the results of one study (*n* = 1) suggest that extraverted personality and agreeableness negatively predicted SVA or led to low levels of SVA (Ling et al., 2021).

### 3.5 Behavioral factors

Behavioral factors (*n* = 10) include short-form video use time and frequency (*n* = 6), past and present time focus (*n* = 1), learning engagement (*n* = 1), academic achievement (*n* = 1), trait mindfulness (*n* = 1), and physical activity (*n* = 2). The results of the study indicate that university students who use short-form video platforms for a long time and with high frequency per day and before bedtime are more likely to be addicted (Hang, 2020; Li et al., 2022; Ling et al., 2021; Qingqing and Liqin, 2021; Yang et al., 2025; Yawen et al., 2020). Focusing on the past and present time has a positive predictive effect on SVA (Liu et al., 2025). Instead, higher learning commitment and academic achievement are less likely to result in a risk of SVA (Quanxi et al., 2024). Physical activity directly correlated with reduced nighttime short-form video use and lower levels of SVA (Yang et al., 2025). Trait mindfulness interventions can also reduce the risk of SVA (Zhang et al., 2025).

### 3.6 Social factors

Social factors (*n* = 11) include social support (*n* = 3), bullying and violence (*n* = 3), interpersonal relationship sensitivity and satisfaction (*n* = 2), and peer influence (*n* = 3). Social support is an individual's perceived support from the external surroundings or expectations (Ding et al., 2024). When social support declines, it can predict the emergence of negative emotions and a decline in life satisfaction, thus directly or indirectly predicting SVA (Ding et al., 2024; Yue et al., 2024; Zhao and Kou, 2024). Bullying refers to social exclusion, repeated attacks, and power imbalances, which can increase the risk of SVA among Chinese university students (Yao et al., 2025; Zhang Y. et al., 2024). Students who experienced violence outside the home have increased odds of mild, moderate to severe SVA by 1.16 times and 1.30 times, respectively (Xue et al., 2025). Sensitivity to interpersonal relationships and low satisfaction can both result in stress, anxiety, and SVA (Hang, 2020; Quanxi et al., 2024). Peer influence mainly refers to the surrounding people's use and behavioral pressure of friends, or the influence of physical environment, which positively predicted SVA (Jianya et al., 2020; Ling et al., 2021; Yue et al., 2022).

### 3.7 Family factors

The impact of family factors (*n* = 5) on SVA, including reciprocal filial piety (*n* = 1), authoritarian filial piety or parenting (*n* = 2), permissive parenting (*n* = 1), behavior pressure of family (*n* = 1), and adverse childhood experiences (*n* = 2). Reciprocal filial piety refers to a close parent-child relationship built through reciprocity, emphasizing that children voluntarily provide emotional and material support to their parents (Fu et al., 2024). Authoritative filial piety or parenting is a non-equal relationship based on the Confucian notion of “Zunzun,” which values children's obedience to their parents' authority (Fu et al., 2024; Ling et al., 2021). Students with authoritarian filial piety or parenting and facing family behavioral pressures are more likely to be SVA (Fu et al., 2024; Yue et al., 2022). In contrast, students with reciprocal piety and permissive parenting are less prone to SVA (Fu et al., 2024; Ling et al., 2021). In addition, adverse childhood experiences can seriously influence university students' addiction to short-form videos. It refers explicitly to parental neglect, abuse, and absence (Xue et al., 2025, 2024).

### 3.8 Motivational factors for media use

Six of the included studies reported motives for media use. Self-compensation motives (*n* = 3) can lead to SVA among Chinese university students (Jianya et al., 2020; Liu et al., 2021; Pei, 2022). Self-compensation refers to the motivation to take specific actions to satisfy the individual's psychological needs and compensate for the dissatisfaction with reality when they are harmed or threatened (Liu et al., 2021). Its motivation can be escapism, pressure relief, soothing negative emotions, and gaining more affirmation and satisfaction (Jianya et al., 2020; Pei, 2022). Short-form video platforms have the characteristics of vertical segmentation, diversification, and interactivity, which satisfy the needs of students for fragmented reading and information acquisition (Jianya et al., 2020; Qingqing and Liqin, 2021; Wang and Zhang, 2024). Media literacy (*n* = 1) refers to an individual's ability to access, interpret, judge, and use media information; lack of media literacy can lead to SVA (Jianya et al., 2020). At the same time, students can use short-form videos to satisfy their needs for emotional expression (*n* = 1), sharing life (*n* = 1), amusement (*n* = 1), unrealistic fantasies of internet celebrities (*n* = 1), and social communication (*n* = 1), all of which are motivations that can lead to SVA (Jianya et al., 2020; Pei, 2022; Qingqing and Liqin, 2021).

### 3.9 Platform-induced factors

Platform-induced factors (*n* = 3) mainly include personalized video recommendation (*n* = 2) and persuasive design (*n* = 2). Personalized video recommendation (PVR) significantly influences users' addiction (Wang and Zhang, 2024; Yao et al., 2025). PVR customizes certain features, products, or services to fulfill users' requirements or interests (Deldjoo et al., 2020). PVR modulates the default mode network in the brain, thereby enhancing attention to content relevance (Yao et al., 2025). Reward areas are also activated, triggering dopaminergic pathways associated with pleasure and reward, further enhancing the video viewing experience and leading to SVA (Yao et al., 2025).

The persuasive design of platforms often aims to get more clicks and monetization from users, which can lead to SVA among university students (Chen X. et al., 2023). Short-form video platforms recommend new videos based on a user's viewing history, integrate purchase links with the video content, and encourage users to order with a single click (Chen X. et al., 2023). During short-video consumption, users experience increased baseline activity in areas related to visual processing, memory, and emotion regulation, leading to addiction (Yao et al., 2025).

## 4 Discussion

### 4.1 Influencing factors through the lens of the I-PACE model

SVA among Chinese university students has emerged as a significant public health concern, reflecting broader anxieties regarding pervasive digital media's psychological and behavioral impacts (Yang et al., 2025; Zhang L. et al., 2024). The university period, corresponding to late adolescence and early adulthood, is a critical developmental stage marked by heightened vulnerability to behavioral dysregulation (Schubert et al., 2017). During this period, career uncertainty and psychosocial transitions may increase reliance on short-form video platforms for emotional escape and stress relief (Guo and Chai, 2024). Excessive use can hinder real-world socialization, impair academic progress, and disrupt emotional regulation and executive functioning (Zhang K. et al., 2023). Against this backdrop, the present systematic review focuses on Chinese university students as a developmentally sensitive group, explores the underlying drivers of their SVA-related behavioral patterns, and intends to inform targeted mental health interventions and future research.

This systematic review provides a comprehensive synthesis of empirical studies examining the influencing factors of SVA among Chinese university students between 2020 and 2025. Drawing on 28 empirical studies retrieved from Chinese and English databases, the findings reveal a high level of SVA among Chinese university students, with TikTok as the most frequently used platform. The regional scope of the study locations is well-represented. Most articles used large sample sizes, a cross-sectional research design, and quantitative research methods to assess the influencing factors of SVA among Chinese university students. However, there is a lack of longitudinal and intervention study designs. The results collectively identify eight categories of influencing factors: demographic, psychological, personality trait, behavioral, social, family, motives for media use, and platform-induced factors.

To provide a unifying lens for interpreting the diverse influencing factors, the present review adopts the I-PACE model (Brand et al., 2019) as a conceptual framework. This model posits that specific person-related variables interact with affective and cognitive responses to influence executive functioning, which in turn shapes addictive behaviors. Within this framework, the eight identified categories of influencing factors can be mapped as follows:

Core factors related to persons include demographic and personality trait factors. In demographic dimensions, female university students, students living in rural areas, who were born in non-only-child families, the lower the grade level, majoring in arts, humanities, sports, and medicine, are more likely to be SVA (Liu et al., 2025; Pan et al., 2024; Xue et al., 2025; Yawen et al., 2020). The higher risk of having SVA in female students may be partly attributable to higher emotional sensitivity and a greater tendency to use short videos for emotional regulation (Wu et al., 2025), which aligns with gender-differentiated patterns of coping and social media engagement observed in previous addiction literature (Soukup, 2015). Meanwhile, students from rural backgrounds or non-only-child families may experience greater social isolation, resource constraints, or familial responsibilities, increasing their reliance on short-form video content as a means of escape or self-soothing (Liu et al., 2025; Pan et al., 2024; Xue et al., 2025). Moreover, students in lower academic years often exhibit less developed self-regulation, media literacy, and academic coping skills, which may render them more vulnerable to compulsive digital behaviors (Quanxi et al., 2024). Similarly, those enrolled in majors with heavier emotional or physical workloads (e.g., medicine, sports) or more open-ended creative exploration (e.g., arts and humanities) may be exposed to higher stress or irregular schedules, which in turn contribute to excessive short-form video use as a form of leisure compensation (Xue et al., 2025; Yue et al., 2022).

In the dimension of personality trait factors, higher levels of neuroticism, shyness, introversion, low core self-evaluation, and boredom may lead to SVA (Liu et al., 2021; Yawen et al., 2020; Zhang L. et al., 2024; Zhang Y. et al., 2024). Neurotic individuals tend to experience more frequent and intense negative emotions, such as anxiety, mood instability, and emotional exhaustion, which may prompt excessive media use as a maladaptive emotional regulation strategy (Abbasi and Drouin, 2019). Similarly, individuals with high levels of shyness and introversion may use short-form videos to avoid real-life social interactions, satisfying their need for connection in a low-risk, asynchronous digital environment (Liu et al., 2021; Yawen et al., 2020). Low core self-evaluation may be more susceptible to digital content's escapist and self-soothing properties, particularly when confronted with academic or interpersonal stressors (Ding et al., 2024). Moreover, boredom proneness—a dispositional tendency to experience boredom easily—has been closely linked to compulsive media use across platforms (Xu et al., 2025). Individuals with high boredom sensitivity often seek immediate stimulation and gratification, making them more vulnerable to short-form video platforms' fast-paced, endlessly scrolling nature (Kohler, 2023).

Social and family factors, as the external environmental factors of persons, influence the individual's SVA. In the dimension of social factors, diminished social support, experiences of bullying, interpersonal sensitivity, low interpersonal satisfaction, and peer influence have been identified as significant predictors of SVA among university students (Ding et al., 2024; Hang, 2020; Jianya et al., 2020; Ling et al., 2021; Quanxi et al., 2024; Xue et al., 2025; Yao et al., 2025; Yue et al., 2024, 2022; Zhang Y. et al., 2024; Zhao and Kou, 2024). Students lacking emotional or instrumental support from peers, family, or institutions may use online platforms for compensatory connection or validation.

In the family factor dimension, students with adverse childhood experiences, authoritarian filial piety or parenting, and facing family behavioral pressures are more likely to be SVA (Fu et al., 2024; Ling et al., 2021; Xue et al., 2025, 2024; Yue et al., 2022). Unhealthy parenting styles can significantly impair students' autonomy, self-esteem, and self-efficacy (La Rosa et al., 2025). These deficits heighten anxiety and weaken effective self-regulation, potentially leading emerging adults to rely on short-form videos as an escape or source of validation, thereby reinforcing SVA risk (La Rosa et al., 2025). These findings underscore the importance of family environment and dynamics in shaping media habits and highlight the need for holistic support systems in university and family contexts.

Affective and cognitive responses include psychological and motivational factors for media use. In the domain of psychological factors, emotional distress—encompassing depression, anxiety, and loneliness—has consistently been identified as a major contributor to SVA (Hang, 2020; Quanxi et al., 2024; Wu et al., 2024; Xu et al., 2025; Xue et al., 2024; Yao et al., 2025; Yue et al., 2024; Zhang L. et al., 2024; Zhao and Kou, 2024). Additional psychological risk variables include elevated levels of life stress, high levels of meaning in life, low subjective wellbeing, intolerance of uncertainty, and reduced use of cognitive reappraisal (Ding et al., 2024; Fu et al., 2024; Ling et al., 2021; Liu et al., 2021; Quanxi et al., 2024; Xue et al., 2025; Yue et al., 2024). These patterns are consistent with compensatory internet use theory (Kardefelt-Winther, 2014), which posits that digital engagement may be a maladaptive coping strategy to alleviate psychological discomfort. Importantly, psychological variables often act as mediators or chained mediators, transmitting the effects of social adversity, family-related stress, platform design, and motivational tendencies on SVA.

In the dimension of motivation for media use, self-compensation motives, information acquisition, lack of media literacy, satisfy needs for emotional expression, sharing life, amusement, unrealistic fantasies of internet celebrities, and social communication, all of which can lead to SVA (Jianya et al., 2020; Liu et al., 2021; Pei, 2022; Qingqing and Liqin, 2021; Wang and Zhang, 2024). Intensive social media use fuels fear of missing out and heightened social comparison, which prompt compulsive content checking (Phuyal et al., 2024). These dynamics align with our observations that unmet social needs and fear of missing out can drive students toward addictive short-form video engagement.

Execution contains behavioral and platform-induced factors. In the behavioral factor dimension, university students who use short-form video platforms for long duration and high frequency every day, before bedtime, who focus on past and present time, are more likely to cause SVA (Hang, 2020; Li et al., 2022; Ling et al., 2021; Qingqing and Liqin, 2021; Yang et al., 2025; Yawen et al., 2020). Excessive screen time, particularly during pre-sleep hours, has been associated with sleep disturbances, delayed bedtime, and impaired academic functioning, further exacerbating reliance on digital platforms as a compensatory behavior (Premalatha, 2024). Frequent passive consumption without purpose, such as “mindless scrolling” or “doomscrolling,” has been linked to decreased self-regulation and elevated psychological distress (Saindon, 2021), further reinforcing compulsive usage patterns. In addition, individuals with a predominant focus on the past or present—rather than future-oriented thinking—tend to exhibit weaker self-regulation and planning ability, increasing their risk of compulsive digital engagement (Liu et al., 2025). Additionally, in terms of platform-induced factors, features like personalized video recommendation and persuasive design are engineered to maximize engagement (Chen X. et al., 2023; Wang and Zhang, 2024; Yao et al., 2025). They create a feedback loop that activates the brain's reward system, making it difficult for users to stop watching.

Furthermore, protective factors against SVA include proactive self-control, resilience, positive cognition, agreeableness, proactivity, extraversion, conscientiousness, emotional orientation, high core self-evaluation, reciprocal piety, permissive parenting, strong learning commitment, academic achievement, and physical activity (Ding et al., 2024; Fu et al., 2024; Jianya et al., 2020; Ling et al., 2021; Liu et al., 2025; Quanxi et al., 2024; Wang and Zhang, 2024; Wu et al., 2024; Yang et al., 2025; Yawen et al., 2020; Yue et al., 2022; Zhang L. et al., 2024; Zhang Y. et al., 2024). Importantly, recent studies suggest that trait mindfulness significantly mitigates SVA by enhancing present-moment awareness and reducing impulsive reactivity to emotional distress (Zhang et al., 2025).

### 4.2 Mediating pathways in the I-PACE model

Within the I-PACE framework, the influencing factors of SVA do not exist in isolation but interact with each other. Numerous studies have identified interactions between persons, affective and cognitive responses that lead to SVA among Chinese university students. More specifically, psychological and personality trait factors are mediating or chained mediating variables for other influencing factors. In terms of psychological factors, emotional distress—including depression, anxiety, and negative affect—exacerbates the effects of social, family, and personality trait factors on SVA. For instance, interpersonal dissatisfaction, bullying victimization, authoritarian filial piety or parenting, and adverse childhood experiences elevate SVA risk by first increasing psychological distress, highlighting the transdiagnostic role of affective dysregulation in behavioral addiction development (Fu et al., 2024; Quanxi et al., 2024; Yao et al., 2025; Yue et al., 2022). Notably, the impact of adverse childhood experiences on SVA can be mitigated through higher resilience and life satisfaction (Xue et al., 2025). Furthermore, Quanxi et al. (2024) found that “meaning in life” partially mediated the relationships between learning engagement and SVA, and between academic achievement and SVA, highlighting the protective role of purpose-oriented cognition in preventing compulsive digital behavior.

Similarly, the interaction between psychological and personality trait factors appears to influence the development of SVA synergistically: individuals with high neuroticism tend to experience anxiety and depression, which undermines self-control and increases dependence on digital content for mood regulation (Zhang L. et al., 2024). The personality trait of shyness enhances the risk of perceived stress on Chinese university students' SVA (Liu et al., 2021). Symptoms of ADHD predict problematic short-form video use through a chain of cognitive reappraisal, emotional distress, and boredom (Xu et al., 2025). Conversely, proactive personality traits reduce addiction risk by enhancing resilience and self-regulatory capacity (Wu et al., 2024).

Personality traits factors, such as deficits in self-regulation and boredom tendencies, mediate or moderate the effects of social, motivational factors for media use, and psychological factors on SVA. For instance, social exclusion and perceived stress contribute to SVA via increased boredom and reduced self-control, with several studies reporting chained mediation effects (Zhang Y. et al., 2024). In another study, perceived stress predicted SVA through self-compensation motivation, with shyness moderating both the direct and indirect pathways (Liu et al., 2021).

Social factors mediate or moderate psychological and personality trait factors on SVA. Yue et al. (2024) further demonstrated that negative cognitive bias elevates the risk of SVA through a sequential mediation pathway involving diminished social support and increased loneliness. In addition, loneliness indirectly increases SVA through reduced social support and lower physical activity levels (Zhao and Kou, 2024). It reveals the interplay between persons and execution.

### 4.3 Inconsistent findings

Despite the comprehensive evidence, several inconsistencies merit further exploration. For example, the role of gender remains inconclusive: some studies reported higher SVA in females, whereas others observed higher usage intensity among males. These discrepancies may stem from differential content preferences, coping motives, or emotional reactivity (Ling et al., 2021; Liu et al., 2021). In terms of grade level, most studies have concluded that the potential for SVA decreases as grade level increases (Pan et al., 2024; Xue et al., 2025). However, one study argued that third-year students are at a higher risk of SVA than first-year students (Quanxi et al., 2024).

Another area of contradiction involves extraversion and agreeableness. While traditionally regarded as protective traits, some studies observed positive associations with SVA. One explanation is that extraverted individuals may be more inclined toward frequent online social interaction, increasing usage time and potential overreliance on digital communication platforms (Ding et al., 2024).

Additionally, the role of depression was inconsistent, with at least one longitudinal study finding no significant predictive effect. It may suggest bidirectional or time-lagged effects, where SVA itself contributes to later depressive symptoms, or vice versa.

The limited diversity of sample characteristics may partially explain conflicting findings regarding demographic, personality, and psychological factors. For instance, gender imbalance, restriction to a single region, specific major or grade level, particular personality traits, or varying levels of depression could all contribute to contradictory results. In addition, most studies employed cross-sectional designs, which may overlook bidirectional or time-lagged effects, further contributing to inconsistencies. These findings highlight the need for research designs and methodologies that consider population heterogeneity more systematically.

### 4.4 Implications for prevention and intervention

Based on the findings, this systematic review proposes several recommendations for preventing and mitigating SVA in Chinese university students. Promoting mental health—enhancing core self-evaluation, resilience, positive cognition, stress management, emotion regulation, and self-control—is essential for coping with and preventing SVA. Enhancing media literacy and encouraging a balanced allocation of time across academic, physical, and entertainment activities is also critical. For students already experiencing SVA, trait mindfulness-based interventions have shown promise. Furthermore, families, educational institutions, and governments should provide supportive environments and implement targeted policies to curb SVA among university populations.

### 4.5 Limitations

This systematic review generated some new findings, but there are also limitations. Firstly, the possibility of missing relevant studies is not excluded. Secondly, this study focused on university students in mainland China, which may limit the applicability of the findings to other countries due to differences in cultural and political contexts. Thirdly, most of the included studies were cross-sectional, meaning that the generalizability of the data is limited by measuring SVA and influencing factor variables at a single point in time (Wu and Zumbo, 2008). Finally, the studies had different research instruments and influencing factors. Therefore, an additional meta-analysis could not be conducted.

### 4.6 Future directions

Future research directions could delve into the policy factor for SVA among Chinese university students. Has the Chinese government introduced relevant policies to intervene in SVA? What is the influence of government policies on SVA in China? It will be a new perspective and a factor that cannot be ignored.

In addition, scholars could adopt more research designs, such as longitudinal and intervention studies, in future studies. More longitudinal studies will provide a better observation of the change process influencing university students' SVA and may be effective in helping resolve the conflicting results of the influencing factors. For instance, using multi-center longitudinal studies, the design could involve recruiting student cohorts from multiple universities across different regions in China, such as eastern coastal, central inland, and western provinces. Participants could be assessed at multiple time points—such as at the start of university, the middle grades, and final year—to track changes in personality traits, psychological, demographic factors, and SVA symptoms.

Intervention studies could provide solutions to the problem of SVA among university students. Randomized controlled trials (RCTs) could be employed to assess the efficacy of various intervention strategies, including digital literacy education, self-control enhancement programs, emotional resilience training, and structured time management interventions. These interventions may be delivered via online platforms or through campus-based psychological services. Embedding these intervention modules into ongoing longitudinal studies would allow for real-time testing of both short-term efficacy and long-term sustainability of behavior change.

## 5 Conclusion

This systematic review highlights the high prevalence of short-form video addiction (SVA) among Chinese university students, and its associations with demographic, psychological, personality, behavioral, social, family, media use motivation, and platform-induced factors. Guided by the I-PACE model, we structured the findings into the person–affect–cognition–execution framework to clarify pathways to SVA. Personal factors include demographic and personality traits. Social and family factors represent external environmental influences on the individual's SVA. Affective and cognitive components comprise psychological factors and motivations for media use. Execution factors include behavioral and platform-induced influences. These components interact with each other and collectively predict SVA throughout university students' development. Psychological and personality trait factors are mediating or chained mediating variables for other influencing factors. Moreover, conflicting demographic, personality, and psychological findings may arise from sample homogeneity and research design limitations, underscoring the need for a multilevel assessment approach.

## Data Availability

The original contributions presented in the study are included in the article/Supplementary material, further inquiries can be directed to the corresponding author.

## Supplementary material

Supplementary File S1 Search equation.
