# Supplementary material for: Influencing factors of short-form video addiction among Chinese university students: a systematic review
Source: Front Psychol. 2025 Sep 22;16:1663670. doi: 10.3389/fpsyg.2025.1663670 (PMC12498229; doi:10.3389/fpsyg.2025.1663670)
Supplement: Supplementary File S1 — Search equation. [file Data_Sheet_1.docx]

CNKI search equation (n=48)

(主题：短视频 + 短视频平台 + 抖音 + 快手) AND (主题：影响因素 + 因素) AND (主题：成瘾 + 依赖+ 过度使用 + 问题性短视频使用 + 沉迷) AND (主题：大学生 + 高校学生）

Wanfang data search equation (n=86)

主题:(短视频 OR 短视频平台 OR 抖音 OR 快手) and 主题:(成瘾 OR 依赖 OR 过度使用 OR 问题性短视频使用 OR 沉迷) and 主题:(影响因素 OR 因素) and 主题:(大学生 OR 高校学生)

Cqvip search equation (n=5)

[((((((题名或关键词=短视频 OR 题名或关键词=短视频平台) OR 题名或关键词=抖音) OR 题名或关键词=快手) AND (题名或关键词=影响因素 OR 题名或关键词=因素)) AND ((((题名或关键词=成瘾 OR 题名或关键词=依赖) OR 题名或关键词=过度使用) OR 题名或关键词=问题性短视频使用) OR 题名或关键词=沉迷)) AND (题名或关键词=大学生 OR 题名或关键词=高校学生))](https://qikan.cqvip.com/Qikan/search/index?LngMySearHistoryIdGuid=d4c42245-ad9a-413f-8c26-645ea68b8f2a&from=Qikan_Article_History)

Web of Science search equation (n=45)

((TS=("short-form video*" OR "short video" OR "Douyin”  OR "Kuaishou” OR "short video application*")) AND TS=("addiction" OR "problematic use" OR "dependence" OR "overuse" OR "indulgence") AND TS=("influencing factor*" OR "influenc*" OR "affect*" OR "impact*" OR "relationship" OR "factor*")) AND TS=("Chinese college student*" OR "Chinese university student*" OR "Chinese undergraduate*" OR "college student*" OR "university student*" OR "undergraduate*")

Scopus search equation (n=47)

( TITLE-ABS-KEY ( "short-form video*" OR "short video" OR "Douyin" OR "Kuaishou" OR "short video application*" ) AND TITLE-ABS-KEY ( "addiction" OR "problematic use" OR "dependence" OR "overuse" OR "indulgence" ) AND TITLE-ABS-KEY ( "influencing factor*" OR "influenc*" OR "affect*" OR "impact*" OR "relationship" OR "factor*" ) AND TITLE-ABS-KEY ( "Chinese college student*" OR "Chinese university student*" OR "Chinese undergraduate*" OR "college student*" OR "university student*" OR "undergraduate*" ) )

Cochrane library search equation (n=15)

#2 (short-form video): ti,ab,kw OR (short video):ti,ab,kw OR (Douyin):ti,ab,kw OR (Kuaishou):ti,ab,kw OR (short video application):ti,ab,kw (Word variations have been searched) 3415

#3 (addiction): ti,ab,kw OR (problematic use):ti,ab,kw OR (dependence):ti,ab,kw OR (overuse):ti,ab,kw OR (indulgence):ti,ab,kw (Word variations have been searched) 139277

#4 (university student): ti,ab,kw OR (college student):ti,ab,kw OR (undergraduate):ti,ab,kw OR (Chinese college student):ti,ab,kw OR (Chinese university student):ti,ab,kw (Word variations have been searched) 19916

#5 (influencing factor): ti,ab,kw OR (influence):ti,ab,kw OR (affect):ti,ab,kw OR (impact):ti,ab,kw OR (relationship):ti,ab,kw 376808

#6 #2 AND #3 AND #4 AND #5 15

PubMed search equation (n=27)

((("short-form video*"[Title/Abstract] OR "short video"[Title/Abstract] OR "Douyin"[Title/Abstract] OR "Kuaishou"[Title/Abstract] OR "short video application*"[Title/Abstract]) AND ("addiction"[Title/Abstract] OR "problematic use"[Title/Abstract] OR "dependence"[Title/Abstract] OR "overuse"[Title/Abstract] OR "indulgence"[Title/Abstract])) AND ("influencing factor*"[Title/Abstract] OR "influenc*"[Title/Abstract] OR "affect*"[Title/Abstract] OR "impact*"[Title/Abstract] OR "relationship"[Title/Abstract] OR "factor*"[Title/Abstract])) AND ("Chinese college student*"[Title/Abstract] OR "Chinese university student*"[Title/Abstract] OR "Chinese undergraduate*"[Title/Abstract] OR "college student*"[Title/Abstract] OR "university student*"[Title/Abstract] OR "undergraduate*"[Title/Abstract])
